# Quality Evaluation of the Ready-to-Eat Avocado cv. Hass

**DOI:** 10.1155/2021/6621449

**Published:** 2021-09-18

**Authors:** Nicole Roberta Giuggioli, Gabriele Chiaberto, Thais Mendes da Silva

**Affiliations:** DISAFA, Università Degli Studi di Torino, Largo Braccini 2, Grugliasco 10095, Italy

## Abstract

Consumer interest in avocado fruit has increased in the last decade in Europe. Nutritional and quality attributes affect the choice of these fruits, whose characteristics must also be maintained in the postharvest period. The preference regarding the feasibility of eating ripe fruits can assure and improve the success of the emerging marketing of avocados. The exposure of fruits to exogenous ethylene (C_2_H_4_) treatment can accelerate the process of fruit ripening. The aim of this work was at improving the existing knowledge about the quality traits of avocado cv. Hass fruits at the ready-to-eat stage. The most important qualitative traits (weight loss, dry matter content, hardness pulp, and external and internal fruit colour) were evaluated up to 96 hours, maintaining the fruit at two different temperatures, T1 (+8°C) and T2 (+17°C). A trained sensory panel was conducted at 96 hours to confirm the quality of avocado cv. Hass ripened with exogenous C_2_H_4_.

## 1. Introduction

Avocado (*Persea americana Mill*) belongs to the Lauraceae family and represents one of the four most important tropical fruits with global production and trade in expansion across Europe. The increase in consumption is related to different factors (the ready availability of the product through various sales channels and its versatility of use and consumption as well as a taste appreciable for the different products) [1]. In particular, its consumption is independent of that of traditional fruits but it is strongly correlated to the *food neophilia* trend. Recently, marketing researches have shown that consumer's preferences are much more affected by lifestyle and fashion trends than economic factors such as income and education [2]. Considering lifestyle changes over the course of a costumer's life, consumer preference and quality requirements are permanently evolving. Therefore, both intrinsic and extrinsic factors are constantly interacting rather than being separate and complementary to each other. Fashion trends and companies' marketing strategies, for instance, repeatedly affect consumer preference to create new food trends and quality standards, which will then result in the formulation of new intrinsic requirements requested by retailers and industries. Avocado can be considered a medicinal fruit due its high antioxidant levels and other nutritional properties [3], and different results regarding consumption of the nutrients in avocado in association with cardiovascular benefits have been reported recently [4]. Healthy properties due the high content of fatty monounsaturated acids, secondary metabolites such as carotenoids and tocopherols, and several bioactive compounds would classify avocado as a superfood. Studies reported that avocado oil is performing thanks to the nutritional and technological characteristics [5], showing stability at high temperatures similar to olive oil [6, 7]. Avocado proteins processed from the oil waste have been shown to have greater emulsifying stability than soy proteins [8] and are therefore suitable for use as functional ingredients in food systems. Then, the use of avocado seed then could have interesting application in the pharmaceutical [9] and food [10, 11] industries. Among the various cultivars, Hass, Arad, Fuerte, and Pinkerton are the main ones commercially known in Europe and the shape and colour of their peels are the main qualitative traits differentiating them. The acceptability of flesh firmness and the consumer intent to purchase are related to the buttery and creamy consistency and, consequently, to the maintenance of high levels of fatty acids [12]. Like other exotic fruits, avocados are transported from the main growing countries (Latin America, the Caribbean, and South Africa) to European markets when they are unripe to avoid injury, product losses, and mechanical damage [13], but to achieve consumer satisfaction, pulp-ripening procedures are required and necessary [14]. As recently reported by Mpai and Sivakumar [15], the time necessary to reach the ready-to-eat stage differs as a function of the variety. The main commercial avocado varieties are “Hass,” “Fuerte,” “Lamb Hass,” “Pinkerton,” and “Ryan,” and the influence of the growing season on their composition and the concentrations of peel epicatechin, phenolics in the pulp, and fatty acids could affect the ripening procedure. The ripening processes in *Persea americana Mill* affect the oil and dry matter (DM) contents, which are inversely related. Different products, such as calcium carbide (CaC_2_), ethylene glycol (C_2_H_5_O_2_), ethylene (C_2_H_4_), methyl jasmonate (C_13_H_20_O_3_), and ethephon (C_2_H_6_ClO_3_P), are commercially available to induce the artificial ripening of climacteric fruit, but it is well known that C_2_H_4_ exposure accelerates softening safely without possible hazards to human health [16, 17]. The stage of maturity at harvest time and the temperature affect the rate of ripening of avocado. The industrial application of the ripening agent with a catalytic generator must be performed in artificial ripening chambers in a range that should be between 10 and 1000 ppm at the optimum temperature of 15.5°C [18]. The time of exposure to the ripening agent is a function of the DM content of avocado that is the most important maturity index for avocado fruits. Avocado with a DM content in the range of 23–26% generally is exposed to a ripening agent in artificial ripening chambers for 1–2 days [19]. Actually, the ripening treatment of avocado fruits is adopted by many picking houses to have *ready-to-eat* fruits, which have shown significant increases in sales by retailers in the market scenario [20, 21]. Generally, *ready-to-eat* means fruits with a high level of service for the consumer (washed, peeled, cut, and packaged), but in some case, such as tropical fruits, they are not processed; this means that they are ready for consumption in terms of ripening but they are not precleaned or cut. The fresh ready-to-eat stage normally describes fruits with a high service level (washed, peeled, and cut) presented at the retail point of sale packaged, but, as in the case of tropical fruits, they can also be displayed at the point of sale whole with the peel and pulp already mature. Previous studies focused their attention on improving the postharvest of fresh avocado fruits by managing the temperature or the use of an edible coating or 1-methylcyclopropene (1-MCP), but limited are those that evaluated the quality of avocado during the ripening stage [22–24]. Nutritional and quality attributes affect the choice of these fruits, whose characteristics must also be maintained in the postharvest period. The eating quality remains the key of the quality concept as it is the baseline for consumer acceptance of fruit before a costumer formulates an idea of preference, and therefore, it is vital for the successfulness of a product. The aim of this work was at improving the existing knowledge about the quality traits of avocado fruits at the ready-to-eat stage. The most important qualitative traits were evaluated up to 96 hours, maintaining the fruit at two different temperatures, T1 (+8°C) and T2 (+17°C). A trained sensory panel was conducted at 96 hours to confirm the quality of avocado cv. Hass ripened with exogenous C_2_H_4_ comparing it with other commercial varieties.

## 2. Materials and Methods

### 2.1. Sampling Procedures and Qualitative Analysis

**Figure 1:**
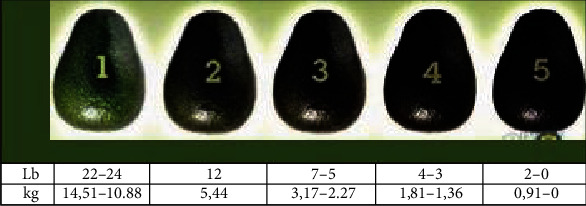
Ripening chart for avocado.

*Persea americana Mill* cv. Hass fruits were imported from Peru according to the storage and transport conditions of one of the most important ripening companies of Northern Italy. Fruits were sampled at the green stage of size 14 (258–313 g). The edible ripeness stage was reached at levels 3 and 4 according to the ripening chart (3.17–1.87 kg of pressure) (Figure 1).

Avocado were experimentally forced to ripen by exposure to C_2_H_4_ (100 ppm) applied at 18°C for 24 h followed by storage at 5°C in a storage room and immediately transported to the laboratory of the University of Turin, Department of Agricultural, Forestry, and Food Sciences (DISAFA). Fruits were stored for up to 96 hours at two different temperatures T1 (+8°C) and T2 (+17°C). For each sample (T1 and T2) and control time (24, 48, 72, and 96 hours), 12 fruits were selected and analysed regarding weight losses, dry matter (DM), skin and pulp colour parameters (*L*, *a*, and b), firmness, and texture profile analysis (TPA). Weight loss (%) was determined using an electronic balance (model SE622), VWR Science Education, Radnor, Pennsylvania, (USA) with a 10^−2^ g accuracy. The weight was monitored for the entire storage time and the loss was calculated as the difference between the initial and final weights.

Dry matter was estimated by drying three replicates of approximately 20 g of material in an oven at 70°C for 24 hours. The fresh and dry weight data were used to calculate the respective DM percentages. Colour measurement was performed in the middle of the peel and pulp using a tristimulus CR-400 chromameter (Konica Minolta, Langenhoven, Germany) according to the Commission International declaring (CIE) *L*^∗^*a*^∗^*b*^∗^ system. *L*^∗^ refers to the lightness and ranged from *L*^∗^ = 0 (black) to *L*^∗^ = 100 (white). Negative and positive values of *a*^∗^ indicate green and red colours, respectively, while positive and values of negative *b*^∗^ indicate yellow and blue colours, respectively.

The firmness and texture profile analysis (TPA) was performed with the Texture Analyser TA.XT. PLUS (Stable Micro Systems, USA) (30 kilo load cell). Since the shape and dimensions of the samples may strongly influence compression tests, the fruits were cut longitudinally into small pieces (3 cm height, 3 cm width, and 3 cm thickness) and each half was laid down and compressed at a pretest speed of 5 mm·s^−1^, test speed of 10 mm·s^−1^, and posttest speed of 10 mm·s^−1^. The distance was set to 8.0 mm, and the trigger force was 5 g.

### 2.2. Sensory Evaluation of *Ready-to-Eat* cv. Hass

#### 2.2.1. Sampling Procedures and Sensory Analysis

After 96 hours of storage at T1 and T2, samples of *ready-to-eat* cv. Hass were evaluated for their sensory properties. Stored fruits were compared with *ready-to-eat* avocado cv. Hass bought directly from the retail point of sale (sample named “competitor”). Two other varieties, Arad and Pinkerton, displayed as *ready-to-eat*, were bought at the same retail point to better proceed with the projective mapping (PM) analysis. In total, a typology of five samples, all from Perù, was considered.

The PM procedure as described by da Silva et al. [25] was applied to verify similarities and differences among samples. Ten panelists—six female and four male—ranging from 22 to 35 years old, from SATA S.r.l. (Alessandria, Italy), with previous experience in sensory evaluation of fresh fruit, were subjected to specific training prior to sensory evaluation. All fruits were cut into halves, divided lengthwise into two pieces, and served to the panelists. Each panelist received four pieces of fruit of each sample codified with a 3-digit code and presented simultaneously, in random order, as requested by the PM procedure. Panelists were asked to score a sensory sheet composed of descriptors using a continuous-intensity scale of 1–9, 1 being “extremely low intensity” and 9 being “extremely high intensity.” The descriptors were chosen based on previous works and included firmness, creaminess, sweetness, bitter, intensity of flavour, intensity of aroma, hazelnut aroma, rancid aroma, and herbaceous aroma.

### 2.3. Statistical Analysis

All the pooled data were analysed using SPSS Statistics 24 (2017, IBM, Milan, Italy) for MAC. Analysis of variance (ANOVA) was performed followed by Tukey's post hoc test, when the differences were significant. Results from the PM analysis were performed with the multivariate multiple factor analysis (MFA). The coordinates *x* and *y* from each assessor of each product were treated as a group of two active variables to build the first two dimensions. Data were not scaled. Furthermore, 95% confidence ellipses were applied around the sample mean points, letting the bootstrap sequence iterate on the assessor's partial (rotated) coordinates instead of the original assessor's data, as suggested by other authors [25]. Using this approach, the confidence intervals do not include the assessor's variability, since the objective is to compare the avocado products. The mean scores obtained for each sample and for each descriptor were used as supplementary variables in the MFA analysis in order to enrich the sample description. Data obtained from the descriptors were classified as continuous and not scaled. A scree plot was made in order to decide how many dimensions to keep. Only variables with a cos2 value higher than 0.25 were plotted in the correlation map in order to select only variables that were significant differentiators of the products.

## 3. Results and Discussion

### 3.1. Qualitative Analysis

The role of water loss in the ripening of avocado cv. Hass was studied by Lallu et al. in 2004 [26] who reported that the water content can initiate rot development. The water content is considered as a maturity indicator, and when avocado matures, the moisture content decreases. The effect of the relative humidity on the water loss and ripening rate of the Fuerte and Hass varieties has been investigated by Adato and Gazit [27]. The authors underlined the negative correlation between the daily rate of water loss from fruits and their ripening index, which increase by up to 40% for avocado with 2.9% water losses. The results reported in Table 1 indicated that the water content of avocado cv. Hass in *ready-to-eat* fruits is still high. For all the samples, water losses increased daily up to 96 hours of storage, but as expected, T2 mainly affects the weight loss content. Avocado fruits maintained at +8°C (T1) can maintain a good level of hydration, thus limiting water losses; at 96 hours of storage, in fact, fruit stored at a low temperature can contain up to six times more water than fruit stored at a temperature of +17°C (T2).

The change in DM content is linked to the fatty acid content, which, in turn, varies among avocado varieties. The dry matter content is well known to be influenced by the respiration rate [28], and with increased ripening, high levels of oil are concentrated in the pulp at the expense of DM [29]. Storage temperatures between 5°C and 10°C are reported to considerably slow down the metabolic activities of avocado fruits, thus slowing down the decrease in dry matter in the same storage period [30]. The results reported in Table 2 confirm those reported in previous studies [30]; in fact, samples maintained at +17°C (T2) have an important decrease showing at the end of the storage time (96 hours) −3.15% loss of DM, compared with the start value (0.87%). For the same storage time, it can be observed that the sample maintained at +8°C (T1) showed a decrease of −1.93% compared with the starting value.

**Figure 2:**
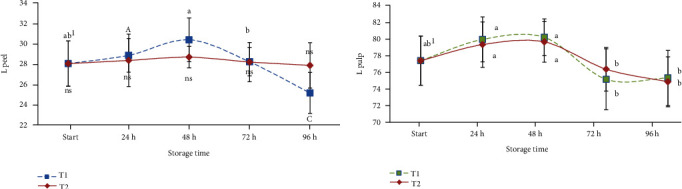
Evolution of the luminosity (*L*) colour parameter of stored cv. Hass (peel and pulp). Values followed by different letters are significantly different at *P* ≤ 0.05 (Tukey's post hoc test).

Different studies report the effect of storage treatments on colour evolution in avocado, and this qualitative parameter is considered, along with the firmness of the pulp, one of the most important in the evaluation of the ripening stage of avocado fruits and the efficiency of the applied technique during the storage period [31–34]. The degradation of chlorophyll and the synthesis of cyanidin 3-O-glucoside are the main factors that promote the browning of the avocado's peel, which also affects the marketability of the fruit. *Ready-to-eat* avocado fruits are characterized by a dark-green to deep purplish colour, but the storage temperature can especially modify the lightness of the fruit. In Figure 2, the evolution of the luminosity parameter (*L*) of the peel and pulp of both samples is reported. At the beginning of storage (start), samples of cv. Hass showed a value of *L* of 27.9. Over time, no statistically significant differences were observed for fruits maintained at +17°C (T2), while the lowest temperature of +8°C (T1) seemed to mainly affect the evolution of the skin colour in terms of brightness. This could be due the higher water content of samples maintained at +8°C as observed in Table 1. At the end of the storage time (96 hours), the losses in the *L* value were greater in the *ready-to-eat* samples, T1 achieving 25.1 compared with 27.8 for samples at T2. Considering instead the *L* value of pulp, the evolution up to 96 hours was similar for both samples stored at the two different temperatures. At the start time, all samples showed 76.9 for the *L* value, achieving 75.0 and 74.6, at T1 and T2, respectively.

**Figure 3:**
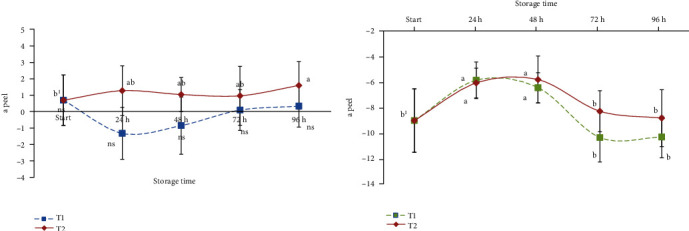
Evolution of greenness (a) colour parameter of stored cv. Hass (peel and pulp). Values followed by different letters are significantly different at *P* ≤ 0.05 (Tukey's post hoc test).

**Figure 4:**
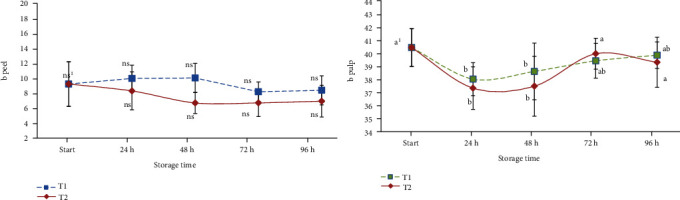
Evolution of the yellowness (b) colour parameter of stored cv. Hass (peel and pulp). Values followed by different letters are significantly different at *P* ≤ 0.05 (Tukey's post hoc test).

The evolution of the greenness (*a*) and yellowness (*b*) of the peel and the pulp is reported in Figures 3 and 4, respectively. Considering the peel of ready-to-eat avocado, no statistically significant differences were observed during the storage period for samples maintained at T1 in terms of *a* and *b* values, while the highest temperature (T2) seemed to influence the green level of the peel with a value of 0.16 (start) to 2.02 at 96 hours. The influence of time at T1 and T2 was similar for the values observed concerning the evolution of the pulp colour (Figure 2), while yellow colour development in pulp seemed to be best maintained by the lowest temperature; in fact, after 96 hours of storage, samples showed a similar colour at the beginning of the shelf-life period (Figure 4).

The texture properties of fruits are strongly related to the judgment and taste evaluation of the final consumer. This expression is very important for avocado fruits, of which texture properties are strongly connected to the content of fatty acids and their distribution within the pulp [35]. Limited data are available on the texture profiles of *ready-to-eat* avocado fruits. In Table 3, some of the most important texture parameters are reported. At both storage temperatures (T1) and (T2), fruit firmness of *ready-to-eat* samples decreased significantly with storage time, although the rate of decrease differed. Samples which demonstrated the highest water losses (T2) also showed the highest hydrolysis of cellulose and hemicellulose, losing 44% of the initial pulp firmness after 96 hours. At the same storage time, samples stored at +8°C (T1) had lost 25% of their initial firmness.

The adhesiveness parameter shows the adhesion of the probe of the instrument used to analyse the sample. Negative values are related to the negative force area measured for the first simulated bite. No statistically significant differences in the time were observed for samples stored at T1. Increasing negative values were observed for T2 samples, which achieved the highest values after 96 hours; this means that the avocado pulp was difficult to remove from the probe due to its pasty and creamy appearance. Gumminess can be considered as the work necessary to disintegrate the sample to a consistency suitable for swallowing, and the decrease in gumminess values was observed to be in agreement with the sample water content loss. Statistically significant differences among samples were already detectable after 24 hours of storage at both temperatures. The resilience parameter measures the elastic recovery of the sample and shares a similar trend with adhesiveness in describing the avocado texture. In fact, no statistically significant differences were observed for samples stored at +8°C (T1). Samples stored at +17°C (T2) showed lowest values and they decreased in time. Statistically significant differences were observed during the storage time.

### 3.2. Sensory Evaluation of *Ready-to-Eat* cv. Hass

To evaluate the quality of avocado cv. Hass ripened and stored after 96 hours, T1 and T2 samples were compared with other *ready-to-eat* commercial varieties. The scree plot suggests that only the first two dimensions should be kept in the analysis as they accounted for almost for 75% of the variance displayed by the samples.

**Figure 5:**
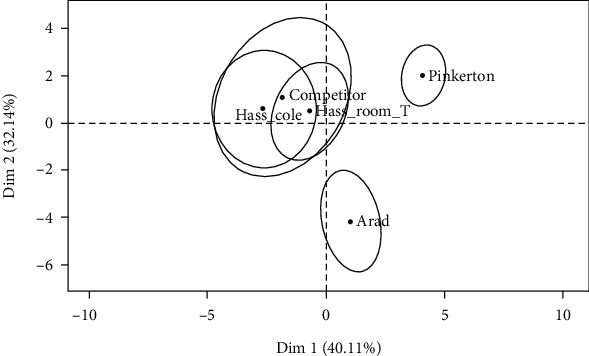
Dimension 1 (Dim 1) and dimension 2 (Dim 2) of the multiple factor analysis individual plot of avocado samples and confidence ellipses.

It is clear that all avocado varieties are widely dispersed, as the confidence intervals displayed by the ellipsis drawn in the MFA map do not overlap (Figure 5). It is also clear that none of the Hass samples (T1, T2, and control) were considered different from each other by the assessors. Hass and Pinkerton samples were positioned on opposite sides when considering the first dimension, and with respect to Arad, the same occurred when the second dimension was taken into account. This means that the quality differentiators among Hass and the two varieties are probably different. In order to gain a deeper understanding considering the quality differences among samples, the sensory descriptors used in the quantitative test need to be plotted.

**Figure 6:**
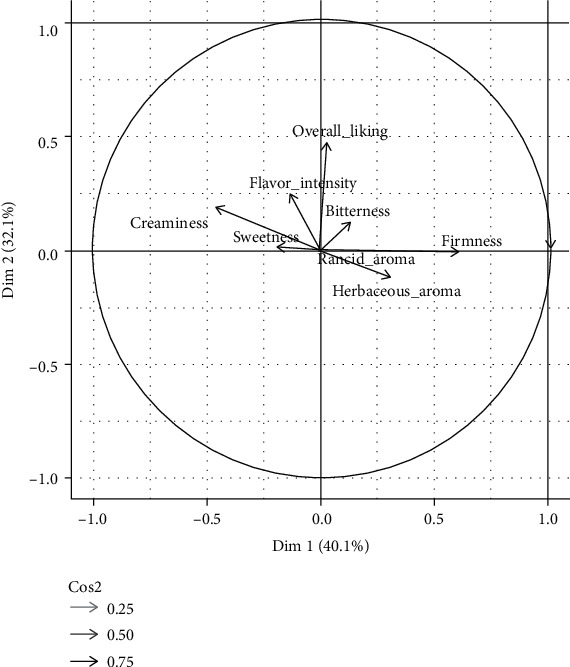
Biplot of sensory evaluation of different *ready-to-eat* avocado varieties, in dimensions 1 and 2.

In Figure 6 it is clear that texture attributes were the most discriminatory, as shown by the higher variance explained by the attribute firmness and creaminess. The PM test confirms that Pinkerton is harder and less creamy than the Hass samples. Pinkerton was also associated with a herbaceous aroma, while the Hass varieties were considered to have a more intense flavour. However, it is important to note that the hazelnut aroma was not displayed in the correlation graphic, despite its common association with the aroma profile of the Hass variety. This means that the samples tasted in this work were not very different from each other considering this important attribute. The overall preference attribute is indicated in a vector close to the Hass and Pinkerton varieties but far away from Arad, indicating that the latter variety was less appreciated by the panel. Probably, this variety was considered tasteless as demonstrated by its opposite position in relation to the flavour intensity attribute.

## 4. Conclusion

Globalization has led to a marked improvement in commercial exchanges, in particular those relating to the fruit and vegetable world, opening the way to new types of products that in the past, it would have been unthinkable to be able to buy in our markets. Consumption of a tropical fruit such as avocado is continuously increasing; this is due to a set of related factors such as the easy availability of the product in the different sales channels and their versatility of use and consumption as well as a taste appreciable for the different products. Among the different fruits, avocado benefits positively from exposure to ethylene to improve the ripening process. The current study was focused on improving the existing knowledge about the quality traits of avocado cv. Hass. Based on physicochemical and organoleptic evaluation, it was found that the cv. Hass can be ripened with ethylene and the fruit quality can be maintained up to 96 hours at +8°C. This new trend is the subject of novel research, especially in the management and the postharvest supply chain, because quality properties of *ready-to-eat* avocado are difficult to monitor considering only the external appearance.

## Figures and Tables

**Table 1:** Weight losses (%) of avocado cv. Hass during storage time.

Samples	24 h	48 h	72 h	96 h
T1	0.20%	0.30%	0.54%	0.57%
T2	0.87%	1.49%	2.19%	3.46%

**Table 2:** Dry matter (DM) content (%) of avocado cv. Hass during the storage period.

Samples	Start	24 h	48 h	72 h	96 h
T1	25.1% ± 0.02	23.73% ± 0.05	23.37% ± 0.03	23.32% ± 0.01	23.17% ± 0.02
T2		22.79% ± 0.02	22.33% ± 0.06	22.15% ± 0.03	21.95% ± 0.01

**Table 3:** Evolution of texture parameters of avocado cv. Hass during storage.

Samples	Storage time				
	Start	24 h	48 h	72 h	96 h
Firmness (*N*)					
T1	3.29 ± 0.39*a*^1^	3.23 ± 0.22*a*	3.10 ± 0.22*ab*	2.78 ± 0.64*ab*	2.46 ± 0.26*b*
T2	3.29 ± 0.39*a*	2.55 ± 0.50*b*	2.40 ± 0.21*bc*	1.95 ± 0.34*c*	1.83 ± 0.34*c*
Adhesiveness (g∗sec)					
T1	−74.4 ± 38.9 ns	−59.1 ± 34.6 ns	−95.1 ± 36.4 ns	−69.4 ± 34.8 ns	−63.2 ± 22.1 ns
T2	−74.4 ± 38.9*ab*	−63.7 ± 38.9*a*	−75.6 ± 39.9*ab*	−104.6 ± 21.8*ab*	−127.1 ± 35.4*b*
Gumminess (g∗sec)					
T1	7250.5 ± 2194.4*a*	5035.3 ± 1952.4*ab*	4921.6 ± 2439.6*ab*	4617.6 ± 2513.8*ab*	3316.7 ± 1263.3*b*
T2	7250.5 ± 2194.4*a*	2082.8 ± 1182.3*b*	2290.1 ± 1126.2*b*	3424.8 ± 1014.1*b*	2593.8 ± 814.5*b*
Resilience					
T1	0.437 ± 0.122 ns	0.309 ± 0.105 ns	0.315 ± 0.084 ns	0.312 ± 0.085 ns	0.288 ± 0.031 ns
T2	0.437 ± 0.122*a*	0.296 ± 0.191*ab*	0.292 ± 0.088*ab*	0.268 ± 0.061*b*	0.257 ± 0.053*b*

Values followed by different letters in the same line are significantly different a *P* ≤ 0.05 (Tukey's post hoc test).

## Data Availability

The data used to support the findings of this study are available from the corresponding author upon request.
